# Glucocorticoid Receptor Activation in Lobular Breast Cancer Is Associated with Reduced Cell Proliferation and Promotion of Metastases

**DOI:** 10.3390/cancers15194679

**Published:** 2023-09-22

**Authors:** Baylee A. Porter, Candace Frerich, Muriel Lainé, Abigail B. Clark, Ishrat Durdana, Jeon Lee, Manisha Taya, Sunati Sahoo, Geoffrey L. Greene, Lynda Bennett, Suzanne D. Conzen

**Affiliations:** 1 Department of Internal Medicine, Division of Hematology and Oncology, University of Texas Southwestern Medical Center, Dallas, TX 75390, USA; 2 Ben May Department for Cancer Research, The University of Chicago, Chicago, IL 60637, USA; 3 Lyda Hill Department of Bioinformatics, University of Texas Southwestern Medical Center, Dallas, TX 75390, USA; 4 Department of Pathology, University of Texas Southwestern Medical Center, Dallas, TX 75390, USA

**Keywords:** glucocorticoid receptor, lobular breast cancer, estrogen receptor, mesothelial clearance, bone metastasis, integrins

## Abstract

**Simple Summary:**

Early-stage invasive lobular breast cancer (ILC) is characterized by multifocal tumor growth in the breast and a distinct pattern of late metastatic spread to other tissues. ILC cells are hypothesized to initiate metastases by exiting the breast via blood and lymphatic channels early in the disease process and then invading and eventually growing in distant tissues. Using in vitro ILC models, this study reveals that expression and activation of glucocorticoid receptor (GR), a stress-hormone-activated nuclear receptor and transcription factor, resulted in inhibition of ILC cell proliferation. Unexpectedly, GR activation also appeared to increase gene expression pathways that promote metastases. Thus, GR may be an important modulator of the low proliferative rate and high late metastatic risk associated with ILC.

**Abstract:**

Estrogen receptor-positive (ER+) invasive lobular breast cancer (ILC) comprises about ~15% of breast cancer. ILC’s unique genotypic (loss of wild type E-cadherin expression) and phenotypic (small individual round cancer cells that grow in discontinuous nests) are thought to contribute to a distinctive pattern of metastases to serosal membranes. Unlike invasive ductal carcinoma (IDC), ILC metastases often intercalate into the mesothelial layer of the peritoneum and other serosal surfaces. While ER activity is a known driver of ILC proliferation, very little is known about how additional nuclear receptors contribute to ILC’s distinctive biology. In ER+ IDC, we showed previously that glucocorticoid receptor (GR) activity inhibits pro-proliferative gene expression and cell proliferation. Here we examined ER+ ILC models and found that GR activation similarly reduces S-phase entry gene expression and ILC proliferation. While slowing tumor growth rate, our data also suggest that GR activation results in an enhanced metastatic phenotype through increasing integrin-encoding gene expression, extracellular matrix protein adhesion, and mesothelial cell clearance. Moreover, in an intraductal mouse mammary gland model of ILC, we found that GR expression is associated with increased bone metastases despite slowed primary mammary tumor growth. Taken together, our findings suggest GR-mediated gene expression may contribute to the unusual characteristics of ILC biology.

## 1. Introduction

Glucocorticoid receptor (GR) is a nuclear hormone receptor involved in physiological and cellular stress responses [1]. GR acts as a potential transcription factor activated by the human stress hormone, cortisol. Hormone activation causes GR homodimerization and translocation to the nucleus followed by transcriptional regulation of stress response genes including cell cycle, cell survival, metabolic, and inflammatory cytokine genes [1]. Recently, the role of GR activation in epithelial tumor cells and the tumor microenvironment has become increasingly appreciated, in part due to the understanding that GR activity has differential effects depending upon cancer subtype [2,3].

Invasive lobular carcinoma (ILC) is a relatively common breast cancer subtype thought to originate from lobular, rather than intraductal, epithelium of the breast. Unlike infiltrating ductal cancer (IDC) morphology which forms cohesive tumor masses, ILC cells demonstrate aberrant E-cadherin expression and often form discohesive multifocal small tumor aggregates in both the primary breast and metastatic sites [4,5]. ILC has a relatively poor pre-operative tumor response rate following anti-estrogen therapy compared to other luminal IDCs, suggesting that estrogen receptor (ER) activity may be a less prominent driver of tumor growth in ILC than IDC [6]. What is also not understood is why early-stage ILC, despite its frequent ER and progesterone receptor (PR) expression and relatively low proliferative rate (<10%), demonstrates a high incidence of late distant metastases [7,8,9,10]. While ILC is ER+/PR+ similar to luminal A IDC, ILC’s pattern of metastatic growth is different [8,9]. A study by Jain et al. examined 1238 patients with breast cancer and compared IDC versus ILC patterns of metastatic disease [11]. They found that IDC recurred most commonly in lung, pleura, and brain, while ILC metastasized more commonly to the bone and peritoneum (*p* < 0.01) [11]. Others have found that compared to IDC, ILC is also significantly more likely to spread to peritoneal surfaces in the abdomen and the retroperitoneum [12,13]. These peritoneal surfaces are composed of a layer of mesothelium supported by connective tissue containing blood and lymphatic vasculature [14]. For ILC to successfully metastasize and invade into the peritoneal serosal layer, ILC cells must “clear” (replace) the mesothelial cells in the peritoneum. The highly morbid sequelae of tumor growth within serosal surfaces (e.g., the peritoneum) can include bowel obstruction, ascites, and pain for ILC patients similar to what is seen in ovarian cancer patients with metastatic disease [12,13].

Recently, our laboratory and others have reported variable GR expression in both ER+ and ER– breast cancers and the effect of GR activity on breast tumor biology [6,15]. For example, we have shown that GR can inhibit tumor cell proliferation in ER+ IDC cell lines by decreasing estrogen-mediated proliferative gene expression and G1/S cell cycle progression, including cyclin D1 (*CCND1*) gene expression [16]. Additional studies in ER- breast cancer suggest that GR activity augments triple-negative breast cancer cell migration, epithelial to mesenchymal transition (EMT), and metastatic cell proliferation in distant organs [17,18]. In this study, we hypothesized that GR expression and activation might contribute to ILC biology by conferring low tumor cell proliferative indices despite ILC’s metastatic characteristics [16].

**Figure 1:**
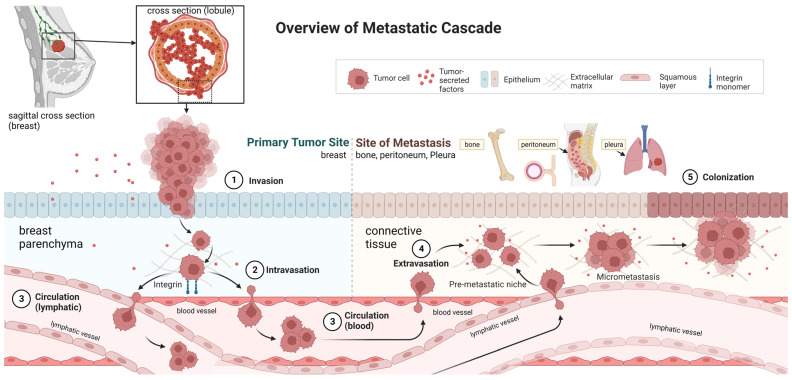
Overview of the ILC metastatic cascade. (1) Local invasion of lobular cancer in situ (LCIS) through the basement membrane into the supporting breast parenchyma is the first step in the ILC metastatic cascade. (2) Intravasation of the ILC cells from breast parenchyma into lymphatics and blood vessels. (3) ILC cell circulation via lymphatics and blood vessels to distant tissues and organs. (4) Extravasation of ILC cells from lymphatics and blood vessels into connective tissue of distal/secondary organ. (5) Further migration and colonization of ILC cells in distant organ tissue, including the serosal layer of the peritoneum, pleura, peritoneal organs, and bone.

To address this hypothesis, we examined GR activity in ER+ ILC SUM44-PE (SUM44) and MDA-MB-134-VI (MM134) cell lines. While we found that GR activation in ILC results in inhibition of G1/S phase cell cycle gene expression and cellular proliferation, GR was also found to upregulate integrin and EMT gene expression. This gene expression was accompanied by increased cell adherence to extracellular matrix (ECM) proteins (a key step in the ILC metastatic cascade) as well as increased mesothelial clearance. In addition, comparison of GR− versus GR+ isogenic SUM44 cells injected into the mouse mammary gland duct revealed larger primary mammary gland tumor volumes from GR− ILC cells and more bone metastases from GR+ ILC cells. Taken together, these observations suggest that GR activation reduces ILC cell proliferation while increasing tumor cell interaction with ECM components, enhancing mesothelial clearance, and promoting metastases (Figure 1).

## 2. Materials and Methods

### 2.1. Cell Lines

SUM44-PE (SUM44) and MDA-MB-134-VI (MM134) ILC cell lines were obtained from American Type Culture Collection (ATCC, Manassas, VA, USA). All cells were authenticated and tested regularly for mycoplasma contamination. SUM44 cell lines were maintained in standard media, Ham’s F-12 (cat# 11765047, Gibco, Grand Island, NY, USA), according to ATCC’s suggested protocol, supplemented with insulin (5 μg/mL, cat# I9278, Sigma-Aldrich, Burlington, MA, USA), transferrin (5 μg/mL, cat# T2252, Sigma-Aldrich), T3 (6.6 ng/mL, cat# T5516, Sigma-Aldrich), ethanolamine (5 mM, cat# E0135, Sigma-Aldrich), NaSe (8.7 ng/mL, cat# S9133, Sigma-Aldrich), HEPES (10 mM, cat# 15630080, Gibco), BSA (0.1%, Sigma-Aldrich), 2% heat-inactivated fetal bovine serum (FBS, cat# 100-1006, GeminiBio, West Sacramento, CA, USA), and 1x penicillin-streptomycin (cat# 15140-122, Gibco). SUM44 cell lines were grown on collagen I-coated tissue culture treated plates. For hormone treatments, cells were cultured with 2.5% charcoal stripped-FBS (made in house from FBS, cat#100-1006, GeminiBio). The MM134 cell line was maintained in standard media according to ATCC’s suggested protocol, L-15/DMEM (cat# 11415064, Gibco; cat# 11965092, Gibco) 50:50 supplemented with 20% FBS and 1x penicillin-streptomycin (cat# 15140-122, Gibco). For hormone treatments, cells were cultured with 20% charcoal stripped-FBS. The Met5A mesothelial cell line was obtained from ATCC and maintained in standard media according to ATCC’s suggested protocol, DMEM high glucose (4.0 mM L-Glutamine, 4500 mg/L Glucose; cat# SH30284.01, Hyclone, Logan, UT, USA) supplemented with 10% FBS, 20 mM HEPES, 1x sodium pyruvate (cat# 11360070, Gibco), 1x penicillin-streptomycin (cat# 15140-122, Gibco). All cells were incubated in 5% CO_2_ at 37 °C.

### 2.2. Recombinant DNA and Stable Cell Line Generation

pLenti-GIII-CMV-GR−GFP-2A-Puro (cat# 321380610395, applied biological materials inc. (abm), Richmond, BC, Canada) and pLenti-GIII-CMV-GFP-2A-Puro (cat# 321380610395, abm) plasmids were transiently transfected into HEK293T cells using the Lipofectamine 3000 kit for lentiviral particle generation. Virus-containing supernatant was used to transduce the target cell line, SUM44. SUM44-GFP (SUM44 GR−) or SUM44-GFP-GR (SUM44 GR+) cell lines were selected with puromycin and regularly sorted for cells with highest GFP signal (ranging from 50–100%) before using for subsequent experiments. Met5a-mCherry-expressing mesothelial cells were generated by lentivirus produced in HEK293 cells transfected with pLenti-EF1a-mCherry, EF1a mCherry was a gift from Oskar Laur (Addgene plasmid #129431; RRID:Addgene_129431, Watertown, MA, USA). Met5A-mCherry-expressing cells were isolated using fluorescence-activated cell sorting (FACS) to generate a stable mCherry-expressing mesothelial cell pool and were regularly sorted for mCherry signal.

### 2.3. Hormones

Dexamethasone (Dex, cat# D2915, Sigma-Aldrich), a synthetic GR ligand, and β-estradiol (E2, cat# E8875, Sigma-Aldrich), a natural ER ligand, were protected from light, solubilized in 100% EtOH, stored at −80 °C, and used at a final concentration (FC) of 100 nM and 10 nM, respectively.

### 2.4. ECM Adhesion Assay

Cells were incubated with charcoal-stripped serum for 48 h followed by treatment with either vehicle (EtOH, 0.1% FC) or Dex (100 nM, FC) for 24 h. Isogenic cell lines SUM44 GR− vs. SUM44 GR+ were incubated in 2% FBS-containing media. For extracellular matrix adhesion assays, pre-coated test strips were used (cat# ECM540, Millipore Sigma, Temecula, CA, USA) according to manufacturer’s instructions. The pre-coated test strips were prepared in a 96-well Nunc plate by incubating with 200 µL 1x PBS without calcium and magnesium (Gibco) for >10 min, then aspirating PBS and allowing wells to dry before adding cells. Cells were then dissociated using dissociation buffer (2 mM EDTA (Pierce Biotechnology, Rockford, IL, USA) in 1x Dulbecco’s PBS without calcium and magnesium) followed by >20 min incubation at room temperature. Cells were collected using dissociation buffer rinsing 2–3 times and then centrifuging at 300× *g* for 3 min at 4 °C. Cells were then resuspended in complete media containing appropriate concentrations of CSS or FBS, and cell counts were measured by cell suspension/trypan blue 1:1 (*v*/*v*) using a Countess 3 Automated Cell Counter instrument (Invitrogen, Carlsbad, CA, USA). The same number of cells was plated in duplicate on ECM protein-coated test strips for each condition. After plating, cells were incubated for at least 1 h in 5% CO_2_, 37 °C. The cell suspension was then removed and washed with 1x PBS containing calcium and magnesium, 3 times (200 µL each wash). Post-washing PBS was removed followed by cell staining and fixation with 100 µL of 0.2% crystal violet solution in 10% ethanol. Staining/fixation buffer was removed, and cells were washed with 1x PBS without calcium and magnesium, 4 times (200 µL each wash). Rinse buffer was aspirated from each well, and cells were subsequently incubated with 100 µL of solubilization buffer for 10 min with constant shaking at 150 rpm at room temperature. Colorimetric quantification was performed by measuring absorbance (540 nm) with a Clariostar plate reader (BMG LABTECH, Ortenberg, Germany). Absorbance measurements were averaged from replicates and normalized to the negative control (BSA) for each of the three independent experiments. A two-way ANOVA was performed using GraphPad Prism version 9 (San Diego, CA, USA).

### 2.5. Cell Proliferation Assay

For cell proliferation assays, single cell suspensions were prepared and measured by using trypan blue/cell suspension (*v*/*v*) 1:1 with a Countess 3 Automated Cell Counter instrument (Invitrogen). Cells were plated in 96-well Sarstedt plates in triplicate. Either SUM44 GR− or SUM44 GR+ cells were plated at 1000 cells/well. MM134 cells were plated at 4000 cells/well. Cells were incubated in medium supplemented with 2.5% (SUM44) or 20% (MM134) FBS for 2 days to allow cells to adhere to plates. Two days (48 h) after plating, FBS-containing media was removed and replaced with media supplemented with 2.5% (SUM44) or 20% (MM134) charcoal-stripped FBS for another 48 h before adding hormone or control treatment, vehicle (0.1% EtOH), E2 (10 nM), Dex (100 nM), or E2 (10 nM) plus Dex (100 nM), FC. A media change with fresh hormone was performed every 3 days. Cells were then incubated for 12 days total. Imaging was performed every 24 h using a Sartorius Incucyte^®^ Live Imaging and Analysis System (Göttingen, Germany). To measure cell proliferation in SUM44 GR− or SUM44 GR+ cell lines, GFP+ cell counts were quantified per image and normalized to day 0, when hormone treatment was initiated. For MM134 cells, relative cell number was quantified per image and normalized to day 0, when hormone treatment was initiated. A two-way ANOVA was performed using GraphPad Prism version 9.

### 2.6. Mesothelial Clearance Assay

The mesothelial clearance assay protocol was adapted from Davidowitz et al. [19] for use with ILC and mesothelial cell lines. Black glass 6-well plates were coated with collagen I according to the manufacturer’s protocol (6 µg/cm^2^, cat# C3867, Sigma-Aldrich) for >1 h at room temperature or 37 °C followed by washing with 2 mL 1x PBS without calcium and magnesium followed by coating with fibronectin (5 µg/mL, cat# F2518, Sigma-Aldrich) according to manufacturer’s protocol for >30 min at room temperature and then washed with 2 mL 1x PBS without calcium and magnesium. Mesothelial monolayer formation: Met5A-mCherry-expressing mesothelial cells were plated at a concentration of 1 × 10^6^ cells/well to allow them to adhere and form a monolayer overnight at 5% CO_2_ in 37 °C. Monolayer formation and retention of mCherry expression was visualized using Incucyte imaging before adding SUM44 spheroids. Spheroid formation/hormone treatments: SUM44-GFP/GFP-GR cells were incubated in media supplemented with 2.5% CSS or FBS for 48 h followed by dissociation with Trype-LE (cat# 12604-013, Gibco), and 400 cells/well were added to a 96-well pre-treated poly-HEMA coated plate (cat# F202003, FaCellitate/Sarstedt, Nümbrecht, Germany) with either normal growth media, vehicle (0.1% EtOH, FC), or Dex (100 nM, FC) for 20–24 h. Spheroid formation was visualized by Incucyte (20–24 h later) and then spheroids were collected from each condition into 15 mL conical tubes before they were added to Met5A-mCherry mesothelial cell monolayers. To prepare mesothelial cells for co-culture, Met5A growth media was removed and washed with 2 mL of 1x PBS followed by addition of 1.5 mL of F12/DMEM 50:50 media supplemented with either 2.5% CSS or FBS for co-culture. SUM44-GFP GR+ or GR− spheroids were then added to separate wells to visualize mesothelial clearance. Live cell imaging parameters: Cells were imaged in a stage top temperature and CO_2_ controlled incubator (37 °C, 5% CO_2_) using an inverted epifluorescence Ti eclipse Nikon microscope with a dry 20× objective at >100 ms exposure using the triggered fast exposure function. Spheroids were imaged every 10 min over the course of 8 h. Quantification: Mesothelial clearance was quantified by normalizing initial SUM44 spheroid size at T = 0 h to mesothelial monolayer mCherry null area at T = 8 h. A minimum of 10 spheroid events were captured (FOV) per biological replicate. A two-tailed Student’s *t*-test was performed using GraphPad Prism version 9.

### 2.7. Immunoblotting

Whole-cell extracts were prepared by lysing cells for 40 min at 4 °C with constant rotation in RIPA lysis buffer (cat# 89900, Sigma-Aldrich), 1x protease and phosphatase inhibitor cocktail set (cat# 78440, Pierce Biotechnology). Total protein concentrations were determined using Pierce 660 nm (cat# 22660, Pierce Biotechnology) protein assays. Electrophoresis and blotting of protein extracts were performed using 4–16% precast Tris-Glycine gradient gels (Bio-Rad Laboratories, Hercules, CA, USA) using mini-PROTEAN tetra handcast and trans-blot turbo transfer systems (Bio-Rad). Primary antibodies used were monoclonal rabbit anti-GR (1:1000, cat# D6H2L, CST, Danvers, MA, USA), Mouse anti-β-actin (1:1000, cat# 8H10D10, CST). Secondary antibodies were goat anti-mouse IgG (H + L) highly cross-absorbed secondary antibody, Alexa Fluor 800 (1:1000, cat# A32730, Invitrogen), and goat anti-rabbit IgG (H + L) highly cross-absorbed secondary antibody, Alexa Fluor 680 (1:1000, cat# A21109, Invitrogen) conjugates. The Chemidot MP imaging system by Biorad was used for immunoblotting detection.

### 2.8. Immunohistochemistry

Tissues were isolated from mice and fixed in 10% formalin for 48 h before transferring to 70% ethanol and embedding in paraffin. Tissue sections were cut at 5 μm per section. Antibodies included rabbit anti-GR (1:500, cat# 3600S, CST), goat anti-luciferase (1:100, cat# AB181640, Abcam, Waltham, MA, USA), rabbit anti-Ki67 (1:400, D2H10, CST), and rabbit anti-pan cytokeratin (1:200, cat# AB9377, Abcam). Imaging: Slides were imaged with a Leica Aperio Versa using the 20× objective. Quantification: For primary mammary gland sections, luciferase-positive cells were quantified using QuPathv0.1.3, positive cell detection function by using cell:OD mean. Object classification training was performed to distinguish between tumor cells (human) and non-tumor cells (mouse) which were identified using luciferase-positive, morphological differences, and robust cell size [20]. Quantification of the Ki67 proliferative index was performed using the positive cell detection function measuring nuclear:OD mean, 1 gland per mouse. A two-way ANOVA was performed using GraphPad Prism version 9 with *p* ≤ 0.05 considered statistically significant.

### 2.9. Animal Studies and Xenograft Mammary Intraductal (MIND) Model

*Cell culture:* SUM44 GR− cells and SUM44 GR+ cells were transduced with luciferin and GFP (pFU-Luc2-Tom) encoding vector via lentivirus at an MOI of 5 [21]. Animal study and injection: All animal studies were approved and performed in accordance with the Institutional Animal Care and Use Committee protocol at The University of Chicago #70899, approval date 12 March 2021. Approximately 500,000 cells of SUM44 GR− L2T or SUM44 GR+ L2T were injected in the mammary duct of the #4 and #9 inguinal glands of 7–8-week old female NSG mice (NOD.Cg-Prkdc^scid^ Il2rg^tm1Wjl^/SzJ) from Jackson Laboratories to establish mammary gland tumors [22]. Mice were imaged with an Xenogen IVIS instrument (Perkin Elmer, Waltham, MA, USA) in the small-animal imaging facility of The University of Chicago. For imaging: Mice were injected with 100 mL of 0.1 M XenoLight D-luciferin (Perkin Elmer cat# 122799) every 2 weeks as described by Lainé et al. [23]. At the end of the study, mice were injected a final time with 200 mL of 0.1 M XenoLight D-luciferin and dissected. Ex vivo peritoneum, lung, liver, bone, and brain were imaged on the Xenogen IVIS instrument (Perkin Elmer, Waltham, MA, USA). Organs were then fixed in buffered formalin 10% for H&E or IHC analysis. Bones were decalcified with Decalcifier 2 (Leica #3800420, Wetzlar, Germany) for 1–2 h until flexible, then rinsed in PBS and stored in ethanol, prior to embedding in paraffin. Graphs and statistical analysis: A two-way ANOVA or two-tailed Student’s *t*-test was performed using Graphpad Prism 9 software, with *p* ≤ 0.05 considered statistically significant.

### 2.10. RNA Sequencing

*Cell treatments:* Cells were treated with charcoal stripped-FBS for 48 h followed by incubation with either vehicle (0.1%, FC), Dex (100 nM, FC), E2 (10 nM, FC), or E2 (10 nM, FC) plus Dex (100 nM, FC) for 6 h before RNA isolation. RNA extraction and sequencing: Total RNA was extracted from cell pellets with Trizol. RNA integrity was determined by the Agilent BioAnalyzer 2100. A TruSeq Stranded mRNA library prep kit (Illumina, San Diego, CA, USA) was used to generate the mRNA libraries. The libraries were analyzed by the Bioanalyzer and multiplexed and sequenced using the NextSeq 500 high output kit (400 M reads) for the libraries at the Next Generation Sequencing Core at the UT Southwestern Medical Center. Raw data from the sequencer were demultiplexed and converted to fastq files using bcl2fastq (v2.17, Illumina). The fastq files were checked for quality using fastqc (v0.11.2) and fastq screen (v0.4.4) [24,25]. Fastq files were mapped to the human hg19 reference genome (from iGenomes, San Diego, CA, USA) using STAR [26]. Read counts were then generated using featureCounts [27]. Rlog normalization and differential expression analysis were performed using DESeq2 [28]. Pathway analysis was performed through Ingenuity Pathway Analysis (QIAGEN IPA, QIAGEN Inc., Venlo, The Netherlands). Genes with more than 1.5-fold change (increase or decrease) and false-discovery rate (FDR) < 0.01 were included in the RNA-seq pathway analysis [29]. Two biological replicates per condition were used.

### 2.11. Quantitative PCR

SUM44 GR+ and MM134 cells were cultured in 2.5% (SUM44) or 20% (MM134) charcoal-stripped FBS for 48 h. Medium was removed, and equal volumes of either vehicle (0.1% EtOH, FC), Dex (100 nM, FC), or hydrocortisone (300 nM, FC) diluted in appropriate media supplemented with 2.5% (SUM44) or 20% (MM134) charcoal-stripped FBS were then added. After 6 h of treatment, 350 μL of lysis buffer (RNeasy Mini Kit, cat# 74104, QIAGEN, Venlo, The Netherlands) supplemented with 2% 2-mercaptoethanol was added to each well to harvest RNA. Total RNA was extracted using the QIAGEN All-Prep DNA/RNA Mini Kit (cat# 74104). cDNA was then reverse transcribed from 0.2 μg of total RNA with Invitrogen SuperScript IV VILO Master Mix (cat# 11756050, Invitrogen) using the MastercyclerX50s (Eppendorf) per the manufacturer’s instructions. The cDNA was diluted in TaqMan Fast Advanced Master Mix (cat# 4444557, Applied Biosystems by Thermo Fisher Scientific, Carlsbad, CA, USA), and quantitative real-time PCR (qRT-PCR) was carried out in a QuantStudio 6 Pro Real-Time PCR System (Applied Biosystems by Thermo Fisher Scientific). The following TaqMan Gene Expression Assays were used: *SGK1*, Hs00178612_m1, FAM-MGB probe; RPLP0, Hs02992885_s1, FAM-MGB probe. Relative quantification of steady-state mRNA transcript expression was calculated according to the standard curve method, as described by Applied Biosystems User Bulletin 2, October 2001, based on the ΔΔ*C*_t_ approach [30]. Transcript levels were normalized to *RPLP0* expression. Each experiment was performed in three technical replicate wells per individual experiment. One representative experiment is shown in figures with error bars representing SEM of the triplicate wells.

### 2.12. Software

Graphs and boxplots were created using Graphpad Prism 9 software for Windows, GraphPad Software, San Diego, CA, USA. Aperio Versa by Leica was used to capture IHC images. QuPathv0.1.3, open-source software was used for IHC analysis and quantification. The Xenogen IVIS instrument (Perkin Elmer, Waltham, MA, USA) was used for bioluminescence imaging of mice and imaging analysis was performed using Living Image 4.7.3. (Perkin Elmer, Waltham, MA, USA). A Clariostar plate reader (Mutli-user SMART Control and MARS data analysis software v6.0, BMG LABTECH GmbH, Ortenberg, Germany) was used to measure absorbance for ECM adhesion assay. A Chemidot MP imaging system by Biorad or a Simple western Jess instrument (bio techne, Minneapolis, MN, USA) was used for immunoblotting detection. Incucyte^®^ Live Imaging and Analysis System was used for cell proliferation. Schematic illustrations were made using Biorender.com, accessed on June 2023.

## 3. Results

### 3.1. GR Activation Attenuates ILC Cell Cycle Gene Transcription and Inhibits Cell Proliferation

To determine whether GR inhibits ER-mediated gene expression pathways (as shown previously in IDC [16]), we first examined global gene expression in two ILC cell lines. We made an ectopically expressing GR cell line (SUM44 GR+) and confirmed GR expression with immunoblotting (Figure S1A). We treated SUM44 GR+ and MM134 ILC cells for six hours with vehicle, E2, Dex, or E2/Dex, and performed RNA sequencing to determine differentially expressed genes (Dex-induced GR activation was confirmed via qPCR of the canonical GR-target gene, *SGK1*, Figure S1B). As expected, using Ingenuity Pathways Analysis (IPA), we found “Estrogen-mediated S phase entry” (a canonical ER-driven proliferative gene expression pathway) to be a top E2-activated pathway in both SUM44 GR+ and MM134 (activation z-score +2.3, +2.0, respectively) (Table 1).

To determine whether GR activation decreases E2-mediated cell cycle gene expression, we also examined the “Estrogen-mediated S phase entry” activation z-scores following combined E2/Dex co-treatment. In SUM44 GR+ and MM134 cells, the activation z-scores were both reduced relative to E2 treatment alone (activation z-score in SUM44 GR+ reduced from +2.3 to +1.41, and in MM134 from +2.0 to +0.8) (Table 1). These results suggest that GR activation inhibits ER-dependent proliferative gene expression. We next examined Dex-induced differential gene expression in the absence of ER activation and found that the GR-mediated activation z-scores were profoundly reduced compared to vehicle treatment alone in both SUM44 GR+ and MM134 cell lines (z-score, −1.6, −1.6 respectively) (Table 1). These results suggest that GR activation in these ILC models also inhibits proliferative gene expression independently of ER activation with E2.

**Figure 2:**
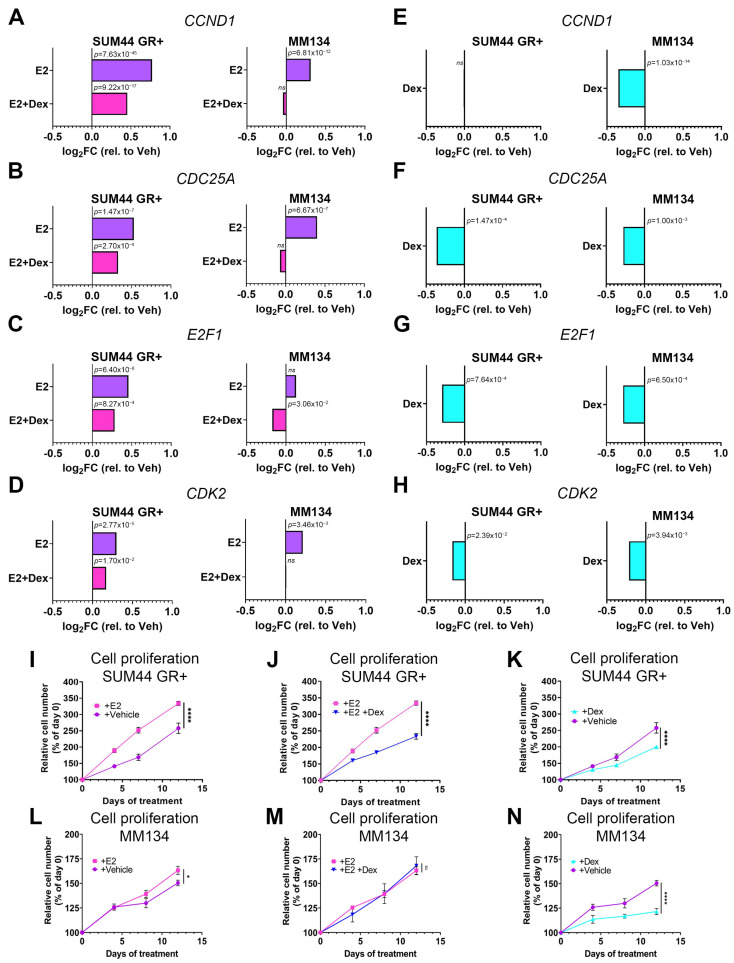
GR activation reduces both proliferation-related gene expression and cell proliferation in ILC cells. SUM44 GR+ and MM134 cells were treated with Vehicle, Dex, E2, or E2/Dex for 6 h and RNA-seq was performed. (**A**) E2 increased gene expression of *CCND1* while addition of Dex reduced *CCND1* gene expression relative to E2 alone. (**B**) E2 increased gene expression of *CDC25A* while addition of Dex reduced *CDC25A* gene expression relative to E2 alone. (**C**) E2 increased gene expression of *E2F1* (SUM44 GR+) while addition of Dex reduced *E2F1* gene expression in both cell lines relative to E2-alone. (**D**) E2 increased gene expression of *CDK2* while addition of Dex reduced *CDK2* gene expression relative to E2 alone. (**E**) Dex reduced *CCND1* gene expression in MM134. (**F**–**H**) Dex treatment reduced gene expression of *CDC25A* (**F**), *E2F1* (**G**), and *CDK2* (**H**). (**I**–**K**) SUM44 GR+ or (**L**–**N**) MM134 cells were treated for 48 h with CSS followed by continuous treatment with Vehicle, Dex, E2, or E2/Dex. (* *p* < 0.05, **** *p* < 0.0001, two-way ANOVA, Šídák post hoc test, *n* = 4 *per group* ±SEM, ≥3 biological replicates). Abbreviations: *CCND1*, Cyclin D1; *CDC25A*, Cell Division Cycle 25A; *E2F1*, E2F Transcription Factor 1; *CDK2*, Cyclin Dependent Kinase 2; E2, estradiol; Vehicle, 0.1% final concentration, EtOH; Dex, Dexamethasone; ns, not significant.

Within the “Estrogen-mediated S phase entry” pathway, we found three of five of the canonical E2-induced cell cycle entry genes (*CCND1*, *E2F1*, and *CDC25A*) were significantly upregulated by E2 in both cell lines (Figure 2A–C, purple bars). Additionally, *CDK2*, an important G1/S phase entry gene, was also significantly upregulated by E2 in both cell lines (Figure 2D, purple bars). While these cell cycle genes were upregulated by E2 alone, they all showed a relative decrease in steady-state expression following combined E2/Dex co-treatment (i.e., GR and ER co-activation). With the exception of *CCND1* in SUM44 GR+ cells (Figure 2E), expression of these genes was also uniformly decreased with Dex treatment alone (Figure 2F–H), consistent with the IPA analysis showing a negative z-score of −1.6 for both cell lines following GR activation alone.

Based on GR-mediated inhibition of G1/S proliferative gene expression, we hypothesized that GR activation would inhibit cell proliferation. To test this with and without E2-mediated ER activation, we treated cells with vehicle, E2, Dex, or E2/Dex and measured cell numbers via Incucyte over 12 days. Results have been plotted separately to distinguish activities of the individual nuclear receptors (Figure 2I–N). We found that SUM44 GR+ cells (Figure 2I) and to a lesser extent MM134 cells (Figure 2L) treated with E2 (ER activation) proliferated more than vehicle treatment (*p* < 0.0001, SUM44 GR+; *p* = 0.047, MM134). Co-treatment with both E2 and Dex (ER and GR co-activation) decreased cell proliferation compared to E2 alone in SUM44 GR+ (*p* < 0.0001, Figure 2J) but did not decrease the modest ER-induced proliferation of MM134 (Figure 2M). Dex treatment alone (GR activation) significantly decreased proliferation of SUM44 GR+ (Figure 2K) and MM134 (Figure 2N) ILC cell lines compared to vehicle treatment (*p* < 0.0001, SUM44 GR+; *p* < 0.0001, MM134). Taken together, these data suggest that GR activation can attenuate ILC cell proliferation through inhibition of cell cycle gene expression.

### 3.2. GR Activation Is Associated with Increased Integrin Gene Expression, EMT Pathway Gene Expression, and Adherence to ECM Proteins in ILC

**Figure 3:**
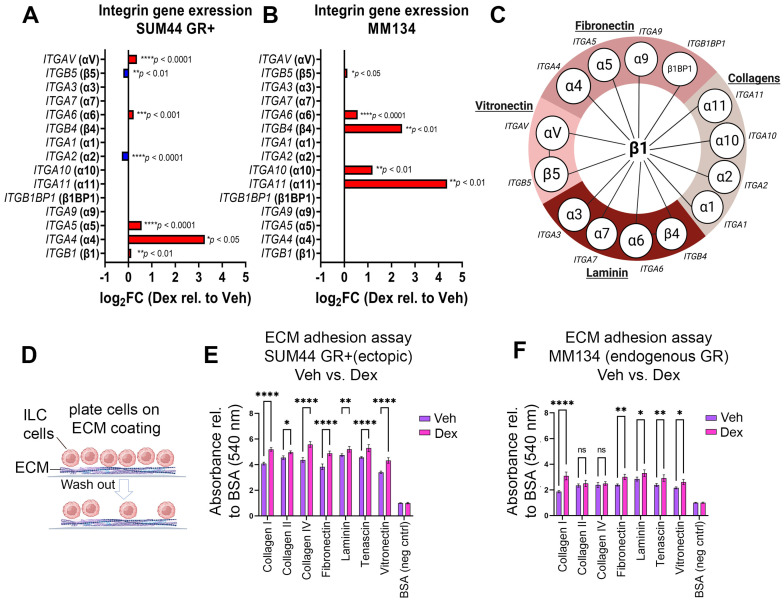
GR activation in SUM44 and MM134 ILC cell lines leads to increased adhesion to ECM proteins. (**A**,**B**) Log 2-fold change of integrin-encoding gene expression of SUM44 GR+ (**A**) or MM134 (**B**) cells treated with Dex vs. Veh (6 h) in CSS. Red and blue bars indicate upregulated or downregulated gene expression, respectively. (**C**) Diagram representing canonical integrin genes and encoded proteins (α/β) organized with respect to their respective ECM ligands (vitronectin, laminin, etc.). (**D**) Schematic of ECM adhesion assay. (**E**,**F**) Adherence measurement by colorimetric assay. (**E**) SUM44 GR+ cells or (**F**) MM134 cells pre-treated with veh (purple) versus Dex (pink) for 24 h. (* *p* <0.05, ** *p* < 0.001, **** *p* < 0.0001, two-way ANOVA, Šídák post hoc test, *n* = 8 per group ±SEM, ≥3 biological replicates). Abbreviations: Veh, Vehicle (0.1% ETOH, FC); Dex, Dexamethasone; CSS, charcoal stripped serum; ECM, extracellular matrix; ns, not significant.

ILC is characterized by a low proliferative rate as well as a “single-file” and discohesive architectural growth pattern considered secondary to loss of the cell surface protein E-cadherin [7]. Despite reduction in ILC cell–cell adhesion, we predicted ILC cells upregulate cell-extracellular matrix (ECM) adhesion proteins required for metastasis and invasion of serosal surfaces [19,31,32]. We therefore wanted to understand whether GR activation altered gene expression for pathways that are critical for cell–ECM adhesion. IPA analysis of SUM44 GR+ and MM134 ILC cell lines showed activation of the “integrin signaling” (z-score, +1.6, SUM44 GR+) and “Epithelial Mesenchymal Transition” (z-score, +0.7, SUM44 GR+; +1.7, MM134) pathways following GR activation by Dex relative to vehicle (Table 1). However, these metastasis-associated pathways showed variable z-scores for E2 and E2/Dex relative to vehicle treatment (Table 1, bottom panel), suggesting that GR activation (rather than ER activation) increases ILC integrin and EMT signaling. We next examined expression of individual integrin genes from the RNA-Seq data and found that SUM44 GR+ cells treated with Dex had increased expression of the integrin-encoding genes *ITGAV*, *ITGA6*, *ITGA5*, *ITGA4*, and *ITGB1* but decreased expression of *ITGB5* and *ITGA2* (FDR < 0.05) (Figure 3A). Treatment of MM134 with Dex led to increased expression of *ITGB5*, *ITGA6*, *ITGB4*, *ITGA10*, and *ITGA11* (FDR < 0.05) (Figure 3B). Overall, these data suggest that GR activation in ILC is associated with increased integrin gene expression and EMT-related pathway gene expression, both of which can contribute to promoting metastasis.

We hypothesized that enhanced expression of integrin genes in ILC would be associated with increased adherence to ECM proteins (Figure 3C). Using, an in vitro assay to measure adhesion of ILC cells to individual ECM proteins (Figure 3D), we found that SUM44 GR+ cells treated with Dex (GR activation) had a significantly increased adherence to collagen, fibronectin, tenascin, and vitronectin compared to vehicle treatment (*p* < 0.0001, Figure 3E). These data are consistent with the increased expression of genes *ITGA4*, *ITGA5*, *ITGA6*, and *ITGAV* (Figure 3A), encoding integrin subunits (Figure 3C). A similar increase was observed in MM134 cell–ECM adhesion for collagen I, laminin, and vitronectin (*p* < 0.0001, Figure 3F). These ECM components serve as substrates for integrins encoded by *ITGA10*, *ITGA11*, *ITGB4*, *ITGA6*, and *ITGB5* (Figure 3B), all of which were increased following GR activation in MM134 cells (Figure 3C). We confirmed the specificity of GR function on increased cell–ECM adhesion (Figure S1C) and the selectivity of Dex-mediated GR activation on cell–ECM adhesion in GR-expressing cells compared to GR-negative cells (Figure S1D). Taken together, our results suggest that GR activation promotes adhesion of ILC cells to ECM proteins in association with increased expression of integrins.

### 3.3. GR Activation in ILC Cells Increases Disruption of a Co-Cultured Mesothelial Layer

**Figure 4:**
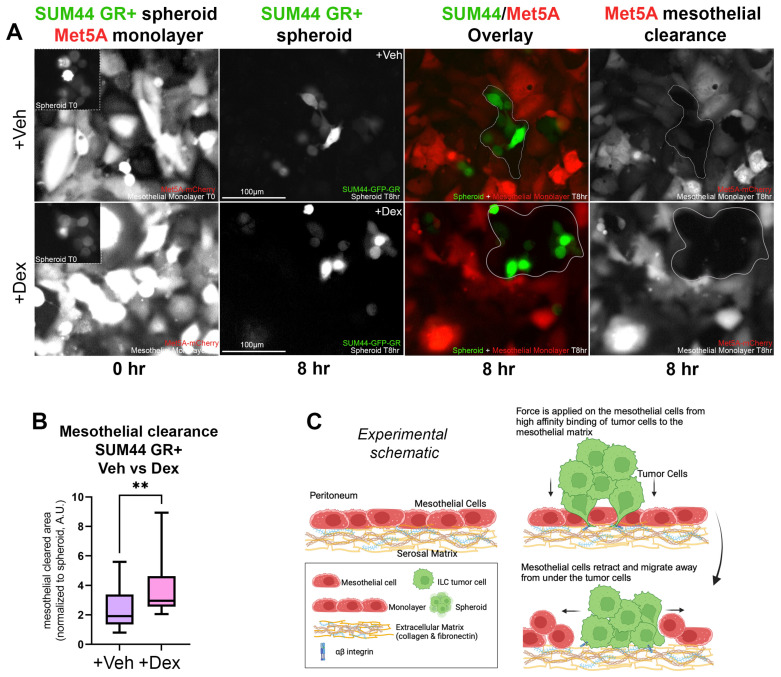
SUM44 GR activation increases mesothelial layer clearance and invasion. (**A**) Clearance of mCherry-labeled Met5A mesothelial cells by SUM44-GR spheroids pre-treated with vehicle (0.1% EtOH, FC) or Dex (100 nM, FC) overnight. ILC cell spheroid size at T = 0 h was used to normalize mesothelial area cleared at T = 8 h. (**B**) Plot of mesothelial area clearance relative to spheroid size (** *p* < 0.01, two-tailed Student’s *t*-test ±SEM, ≥3 biological replicates, ≥20 spheroids per condition). (**C**) Experimental schematic (created with biorender.com) of mesothelial clearance assay as first described in [19,31]. Abbreviations: Veh, Vehicle (0.1% ETOH, FC); Dex, Dexamethasone; T0, timepoint 0 h; T8, timepoint 8 h.

Compared to IDC, metastatic ILC cells are more likely to invade and colonize serosal layers such as the pleura, peritoneum, and retroperitoneum [16,33,34,35,36]. As shown previously in ovarian cancer cell models, E-cadherin-negative tumor cells are more likely to successfully invade a mesothelial monolayer [19,31]. Based on the association of GR activation with higher integrin and EMT pathway gene expression in our ILC models, we next asked whether GR expression in ILC cells would result in increased clearance of the mesothelial layer. We first tested SUM44 GR+ spheroids pretreated with or without Dex in the mesothelial clearance assay (Figure 4A). Using green-labeled SUM44 GR+ cells and red-labeled Met5A-mesothelial cells, layered on top of fibronectin and collagen I, we observed enhanced intercalation and spreading of the Dex-treated SUM44 GR+ spheroids between and underneath mesothelial cells (Figure 4A). Dex-treated SUM44 GR+ cells cleared significantly larger areas of the mesothelial monolayer when compared to vehicle (*p* < 0.001, two-tailed Student’s *t*-test) (Figure 4B). In addition, comparison of mesothelial clearance between SUM44 GR− versus GR+ cells in steroid-containing fetal bovine serum (FBS, Figure S1E) showed that GR+ cells had significantly larger areas of mesothelial monolayer clearance compared to GR− cells (*p* < 0.0001, two-tailed Student’s *t*-test) (Figure S1F). Our data (Figure 4A) demonstrate that GR activation enhances the ability of ILC spheroid cells to adhere and intercalate through a mesothelial monolayer, displacing the mesothelial cells from the ECM (fibronectin and collagen I) as described for ovarian cancer cells by Iwanicki et al. (2011) (Figure 4C) [31]. More efficient displacement of the mesothelial layer is consistent with a potentially greater ability for GR+ ILC cells to colonize and metastasize successfully in serosal surfaces.

### 3.4. GR Expression Is Associated with Decreased Primary Tumor Growth and Increased Bone Metastases in an ILC Xenograft MIND Model

**Figure 5:**
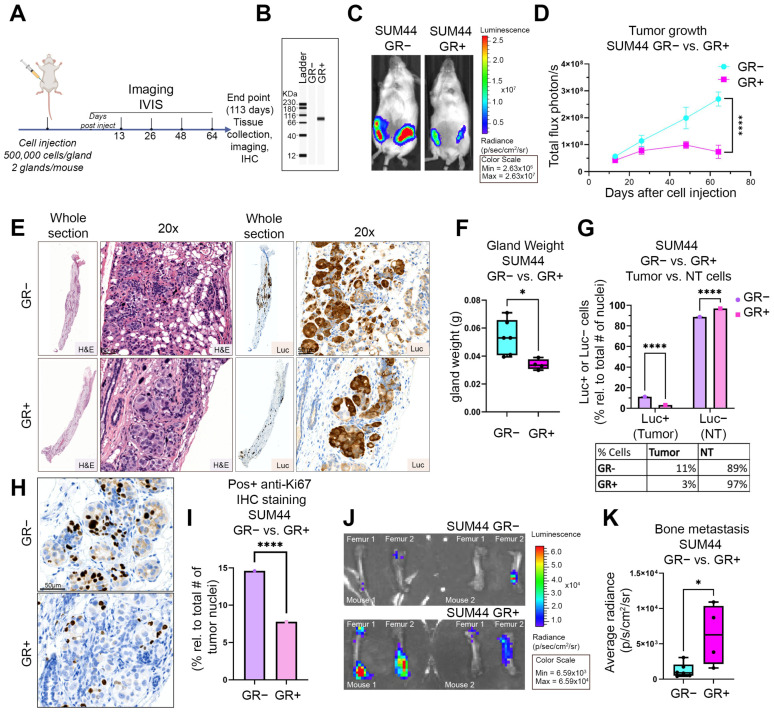
GR+ ILC xenografts demonstrate decreased primary tumor volume and increased distant metastasis to bone. (**A**) Experimental design for MIND model. Mice were imaged by IVIS 13, 26, 48, 64, and 113 days after nipple injection of SUM44 GR− or GR+ cells. (**B**) Western blot of GR expression for SUM44 GR− and SUM44 GR+ cells pre-injection. (**C**) Representative in vivo luminescence of SUM44 GR− versus GR+. (**D**) Bioluminescence signal from primary GR− (*n* = 6) vs. GR+ (*n* = 4) tumors (**** *p* = 0.0001, two-way ANOVA, Šídák post hoc test, *n* = 4 per group ±SEM, ≥2 biological replicates). (**E**) Representative histological images of primary tumor sections in GR− and GR+ H&E (left) and anti-luciferase IHC (right). (**F**) Gland weight (in grams) of SUM44 GR− (*n* = 3) versus GR+ (*n* = 2) (* *p* = 0.016, two-tailed Student’s *t*-test ±SEM, *n* ≥ 2 biological replicates). (**G**) Mammary gland tumor (luc+) vs. non-tumor cells (luc–, NT) in SUM44 GR− (*n* = 3) versus GR+ (*n* = 2) (**** *p* = 0.0001, two-way ANOVA, Šídák post hoc test, *n* = 6 per group ±SEM, ≥2 biological replicates). (**H**) Representative Ki-67 IHC of SUM44 GR− (*n* = 3, left) and GR+ (*n* = 2, right) tumors quantified in (**I**) (**** *p* = 0.0001, two-way ANOVA, Šídák post hoc test, ±SEM, ≥2 biological replicates; # = number). (**J**) Ex vivo luminescence of SUM44 GR− (*n* = 6) mouse femurs versus GR+ (*n* = 4) femurs, quantified in (**K**) (* *p* = 0.022, two-tailed Student’s *t*-test ±SEM, ≥2 biological replicates). For the original image of the Western blot, please see the File S1.

Because we found that GR activation decreased ILC cell proliferation in vitro, we next examined whether GR expression would reduce primary tumor growth in vivo. We implanted SUM44 GR+ or GR− luciferase-expressing cells through the nipple of NSG mice using the Mammary INtraDuctal (MIND) model, followed by in vivo bioluminescent imaging of the mice every 2 weeks for up to 113 days post injection (Figure 5A) [22,37,38]. We confirmed differential GR expression in the two isogenic cell lines using Western blotting immediately prior to implantation (Figure 5B). Bioluminescence radiance was significantly different between SUM44 GR+ and GR− tumors at day 64 (representative images shown in Figure 5C). We observed that SUM44 GR+ tumors exhibited significantly less growth over the course of 64 days post-implantation compared to SUM44 GR− (*p* < 0.0001, two-way ANOVA with Šídák post hoc test) (Figure 5D). NSG mice were then followed with minimal handling until 113 days after implantation. At this point, mice had to be sacrificed due to IACUC protocol guidelines and all organs and femurs were harvested for subsequent IHC analysis and ex vivo bioluminescence. A subset of mammary glands (*n* = 2, GR+ and *n* = 3, GR−) were fixed and embedded in paraffin for H&E staining and IHC analysis. At the end of the study, we noted that there was a reduction in anti-GR antibody IHC staining (Figure S2A) compared to initial western blot analysis of GR+ SUM44 cells immediately before injection (Figure 5B). Therefore, we also used anti-luciferase antibody positive staining as a secondary method for identification of human ILC xenografted cells. H&E staining and anti-luciferase antibody IHC confirmed that SUM44 GR− cells developed larger mammary gland tumors compared to SUM44 GR+ cells (Figure 5E). In addition, all mammary glands were weighed at sacrifice and SUM44 GR− (*n* = 6) glands had significantly increased weight compared to SUM44 GR+ (*n* = 4) mammary glands (*p* = 0.016, two-tailed Student’s *t*-test) (Figure 5F). Using luciferase staining, we distinguished cancer cells (human) from non-tumor cells (mouse) and quantified luciferase-positive cells (tumor) compared to luciferase-negative cells (NT) (Figure 5G). Mammary glands injected with SUM44 GR+ cells had a significant relative decrease (73%) in percentage of cancer cells compared to those injected with SUM44 GR− cells (3% vs. 11%, *p* < 0.0001, two-way ANOVA with Šídák post hoc test) (Figure 5G). Additional IHC staining with anti-Ki67 antibody revealed increased positivity of SUM44 GR− tumors (Figure 5H, top panel) compared to SUM44 GR+ tumors (Figure 5H, bottom panel). Quantification of Ki67 positive tumor cells showed that SUM44 GR− tumors had a two-fold relative increase in percent positivity compared to SUM44 GR+ tumors (14% vs. 7% *p* < 0.0001, two-way ANOVA with Šídák post hoc test) (Figure 5I). In summary, GR expression in SUM44 ILC cells is associated with decreased ILC primary tumor burden and proliferation.

Based on GR-mediated increase of integrin and EMT IPA pathway activation, as well as ECM adhesion and mesothelial clearance, we next examined metastasis. We hypothesized that GR expression would lead to increased metastasis. Ex vivo bioluminescence imaging (Figure 5J) and quantification (Figure 5K) showed that xenografted SUM44 GR+ cells compared to GR− cells resulted in an increased burden of bone metastases (*p* = 0.022, two-tailed Student’s *t*-test). There was no statistically significant difference in the burden of peritoneal metastasis (Figure S2B,C) or any other organs from mice implanted with SUM44 GR+ or GR− cells, which may be due to limited sample size. Based on these observations in xenografted SUM44 GR+ versus GR− ILC, we conclude that GR expression is associated with decreased primary tumor growth and increased bone metastases, consistent with the GR-mediated gene expression studies (Table 1) showing reduced cell cycle and increased integrin and EMT pathway activation.

## 4. Discussion

In this study, we evaluated two ER+, E-cadherin negative, ILC cell lines that recapitulate many of the phenotypic characteristics commonly seen in clinical ILC. Similar to our previous findings in luminal A infiltrating ductal cancer model cell lines MCF7 and T47D cells, we found that GR activation inhibited cell cycle gene expression (*CCND1*, *CDC25A*, and *E2F1*) with concomitantly decreased cell proliferation. Gene expression analysis also revealed that several integrin-encoding genes were upregulated following GR activation, consistent with the ability of some ILCs to readily invade serosal surfaces, including the peritoneum and retroperitoneum. Using an ECM adhesion assay, we found that GR activation increased ILC cell adherence to all ECM proteins, a requisite for cancer cell invasion into serosal layers. Using a well-established in vitro assay developed by the Brugge Lab [31] that measures mesothelial layer invasion by tumor cells, we found that GR activation in ILC cells enhanced mesothelial monolayer disruption. In vivo, GR-expression in SUM44 cells also increased metastatic burden, most notably to the bone.

Unique features of ILC have recently been highlighted by molecular biologists, cancer biologists, and pathologists [9,10,15,39]. For example, ILC has unusual features that include a generally low proliferative rate and a high propensity for clinically relevant late metastasis. The molecular underpinnings of these characteristics have been postulated to be secondary to the loss of E-cadherin function, as well as activating mutations in HER2, PI3K, and P53 within the setting of tumor evolution and metastasis [16,34,40]. Our laboratory previously observed that GR activation is associated with inhibited IDC proliferation and with improved relapse-free survival [16,34,40]. While GR has been shown to inhibit ER-mediated gene expression previously, our data here suggest that GR appears to inhibit estrogen-independent cell cycle progression similar to what has been previously described for osteosarcoma and hepatoma cell lines [41]. Previous data have shown that GR activation in tenocytes can cause senescence, and we also observed GR-mediated enrichment of the IPA senescence gene expression pathway in our ILC cell lines [42,43]. Mechanistic experiments examining the role of GR in cell cycle inhibition and senescence pathway activation are underway. Both of these properties could contribute to late development of metastases in ILC.

EMT allows for cellular plasticity and is regulated by transcription factors that promote mesenchymal rather than epithelial cell characteristics [18,44]. In breast cancer, EMT activation is associated with single cell or collective cell migration into surrounding breast parenchyma and associated blood and lymphatic vessels [18,45]. Previously it was shown by Liu et al. that GR activation can induce an EMT program through downregulation of E-cadherin (*CDH1*) and upregulation of vimentin (*VIM*) gene expression in pancreatic adenocarcinoma giving rise to increased cellular motility [46]. These changes also led to a more invasive phenotype, similar to our observations in GR+ ILC. In ILC cell lines, we found that GR activation increased EMT gene expression, including *FGF*, *MAPK3K1*, *IL6R*, *JAK2*, *SMAD3*/*NFKB2* (SUM44), and *CDH2*/*STAT3*/*YAP1* (MM134). Similar to pancreatic adenocarcinoma where *CDH1* (E-cadherin) is downregulated, GR activation in ILC may act as a complementary pathway to E-cadherin loss by upregulation of EMT gene expression and induction of a metastatic phenotype [46]. How GR acts upstream of multiple pathways to induce the metastatic cascade is under active investigation.

The peritoneum is often a site of distant metastasis in ILC, causing extensive morbidity and posing an important clinical challenge. Previous models of ovarian tumor cell-mesothelium interaction showed the cancer cells generated integrin-dependent forces to clear mesothelial cells [31]. In contrast to ILC, ovarian cancer is known to shed directly from the fallopian tubes and ovary into the peritoneal cavity, while ILC likely travels through the lymphatics and blood vessels to invade into the mesothelium and subsequently into serosal tissues. Iwanicki et al. suggested that ovarian cancer spheroids use α5β1 integrins to dissociate fibronectin during intravasation of the mesothelial monolayer [31]. Genes encoding α5β1 integrins were upregulated in Dex-treated SUM44 GR+ ILC cells that also displayed increased mesothelial clearance. Taken together, these data suggest that GR-mediated integrin expression in ILC cells might induce mechanical dissociation of fibronectin, similar to what is seen in ovarian cancer models [31]. The metastatic tropism of MM134 and SUM44 could be due to GR-induced cell-specific integrin expression. The increased ability of these cells to undergo mesothelial clearance has direct implications for successful metastasis, invasion, and seeding of the serosal surfaces and likely, bone marrow and bone cortex (Figure 1).

Our data also found increased metastatic bone burden in mice engrafted with SUM44 GR+ ILC cells compared to GR− cells. Few studies have examined the direct role of GR in ILC bone metastasis. However, recent evidence showed that activation of serum and glucocorticoid-inducible kinase 1 (*SGK1*), a canonical target gene of GR, promoted bone metastasis, and inhibition led to reduced bone metastatic burden [47]. Additionally, creation of the pre-metastatic niche is an important step in establishing distant metastases to bone and is associated with increased levels of osteoclastogenic cytokines such as TNF-α and IL-6 [48,49]. We found that *TNFA* and *IL6* expression was upregulated in SUM44 GR+ tumor cells, suggesting a possibly enhanced capacity to metastasize to the bone. Future studies will include studying selective GR mixed agonists/antagonists in ILC syngeneic and xenografted mouse models to evaluate effects on both primary tumor growth and metastasis.

## 5. Conclusions

Here we found that GR activation in ILC cell line models decreases both ER-mediated and ER-independent cell cycle gene expression and is associated with slowed ILC cell proliferation. GR activation also upregulates integrin and EMT gene expression pathways, and is associated with increased ECM protein adherence, and enhanced mesothelial clearance in a serosal (e.g., peritoneum) surface model. Furthermore, we find that GR expression in ILC cells is associated with decreased in vivo primary tumor growth and increased metastasis to bone and potentially serosal tissues.

## Figures and Tables

**Table 1:** Canonical IPA pathway analysis of ER, GR, and combined ER/GR activation in ILC cell lines.

Ingenuity Pathway Analysis		Activation z-Score			*p*-Values
		E2 vs. Veh	E2 Plus Dex vs. Veh	Dex vs. Veh	
**Cell proliferation**					
Estrogen-mediated S phase entry	SUM44	+2.3	+1.4	−1.6	4.79 × 10^−6^, 4.47 × 10^−3^, 3.09 × 10^−4^
	MM134	+2.0	+0.8	−1.6	4.79 × 10^−3^, 4.23 × 10^−2^, 1.02 × 10^−4^
**ECM adhesion (metastasis)**					
Integrin signaling	SUM44	−1.5	−0.2	+1.6	1.15 × 10^−4^, 5.13 × 10^−6^, 5.75 × 10^−3^
	MM134	ns	+0.2	ns	1.7410 × 10^−5^
Epithelial Mesenchymal Transition	SUM44	−1.4	+0.5	+0.7	1.05 × 10^−5^, 5.89 × 10^−5^, 1.02 × 10^−4^
	MM134	+1.1	+1.6	+1.7	2.29 × 10^−4^, 1.82 × 10^−3^, 1.41 × 10^−2^

Note: SUM44 GR+ and MM134 cells were treated with Vehicle (0.1% EtOH, FC), Dex (100 nM, FC), E2 (10 nM, FC), or E2 (10 nM, FC) plus Dex (100 nM, FC) for 6 h. Global gene expression was assessed followed by differential gene expression comparing E2, E2/Dex, and Dex to vehicle and evaluation with Ingenuity Pathway Analysis (IPA). Abbreviation: ns, not significant.

## Data Availability

Dataset publicly available, GSE241994; GSE241992; GSE241993.

## Supplementary Materials

The following supporting information can be downloaded at: https://www.mdpi.com/article/10.3390/cancers15194679/s1, Figure S1: (A) Immunoblot detection of GR expression across ILC cell lines MDA-MB-134-VI (MM134), SUM44, SUM44 ectopically expressing GR, and control cell line MCF7. (B) Quantitative PCR analysis of GR *activation* by Dex or hydrocortisone (cortisol) for the canonical GR target gene, *SGK1.* (C) Extracellular matrix adhesion assay comparing SUM44-GR− vs. SUM44-GR+ incubated in FBS. (D) SUM44-GR− cells have decreased adherence to ECM proteins after treatment with Dex and vehicle compared to GR+ cells. (E) Mesothelial clearance assay of Met5a-mcherry and SUM44 GR− vs. GR+ cells labeled with GFP. (F) Mesothelial clearance was measured using T0 spheroid size and normalized to mcherry null area T8h. Figure S2. (A) H&E, luciferase and GR IHC of GR− (*n* = 3) and GR+ (*n* = 2) representative primary tumors 113 days after intraductal injection SUM44 cells showing increased primary tumor size and distinct proliferative and invasive pattern of GR− tumors. (B) Average radiance of peritoneum metastasis in SUM44 GR− and GR+ MIND models. (C) Representative images of *ex* vivo bioluminescence of luciferase signal from peritoneum metastasis. File S1: Original image of Western blot.

## Abbreviations

Abbreviation	Definition
ER/ER+	Estrogen receptor (positive)
ILC	Invasive or infiltrating lobular carcinoma
IDC	Invasive ductal carcinoma
GR/GR+/GR−	Glucocorticoid receptor (+ = positive; − = null)
PR/PR+	Progesterone receptor (+ = positive)
EMT	Epithelial to mesenchymal transition
SUM44	SUM44-PE, endogenously GR null
MM134	MDA-MB-134-VI, endogenous GR positive
ECM	Extracellular matrix
E2	Estradiol, used at 10 nM is an estrogen receptor ligand which induces estrogen receptor transcriptional function
Dex	Dexamethasone, used at 100 nM. Is a high affinity synthetic glucocorticoid receptor ligand which induces glucocorticoid receptor transcriptional function
E2/Dex	Combination of 100 nM dexamethasone treatment and 10 nM Estradiol treatment to induce concurrent GR and ER transcriptional programs
VC	Vehicle control, 100% ETOH
CSS	Charcoal stripped FBS used to deplete cell media of hormone mediated activation
FBS	Fetal bovine serum
GFP	Green fluorescent protein with excitation at 488 nm
mCherry	Monomeric red fluorescent protein derived from Discosoma sp. with excitation at 587 nm
IPA	Ingenuity pathway analysis
T	Time
hr	hours
Luc+/−	Anti-luciferase antibody positive (+) or (−) staining
FC	Final concentration
