# Supplementary material for: Glucocorticoid Receptor Activation in Lobular Breast Cancer Is Associated with Reduced Cell Proliferation and Promotion of Metastases
Source: Cancers (Basel). 2023 Sep 22;15(19):4679. doi: 10.3390/cancers15194679 (PMC10571671; doi:10.3390/cancers15194679)
Supplement: Supplementary file 1 [file cancers-15-04679-s001.zip › cancers-2557328-Supplemental Figures.pdf]

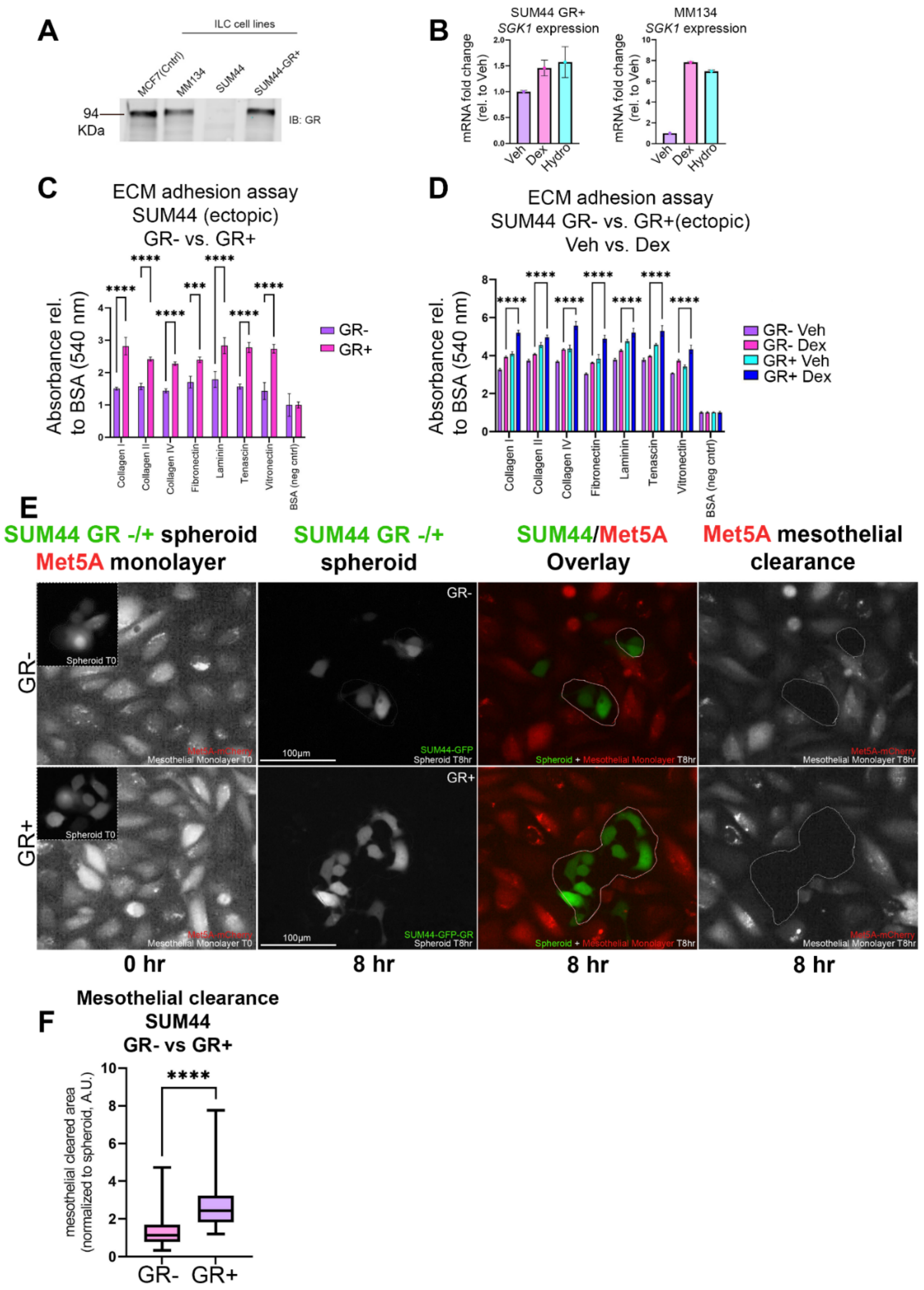

Figure S1. A) Immunoblot detection of GR expression across ILC cell lines MDA-MB-134-VI (MM134), SUM44, SUM44 ectopically expressing GR, and control cell line MCF7. B) Quantitative PCR analysis of GR *activation* by Dex or hydrocortisone (cortisol) for the canonical GR target gene, *SGK1.* C) Extracellular matrix adhesion assay comparing SUM44-GR - vs SUM44-GR+ incubated in FBS. D) SUM44-GR- cells have decreased adherence to ECM proteins after treatment with Dex and vehicle compared to GR+ cells. E) Mesothelial clearance assay of Met5a-mcherry and SUM44 GR- vs GR+ cells labeled with GFP. F) Mesothelial clearance was measured using T0 spheroid size and normalized to mcherry null area T8h.

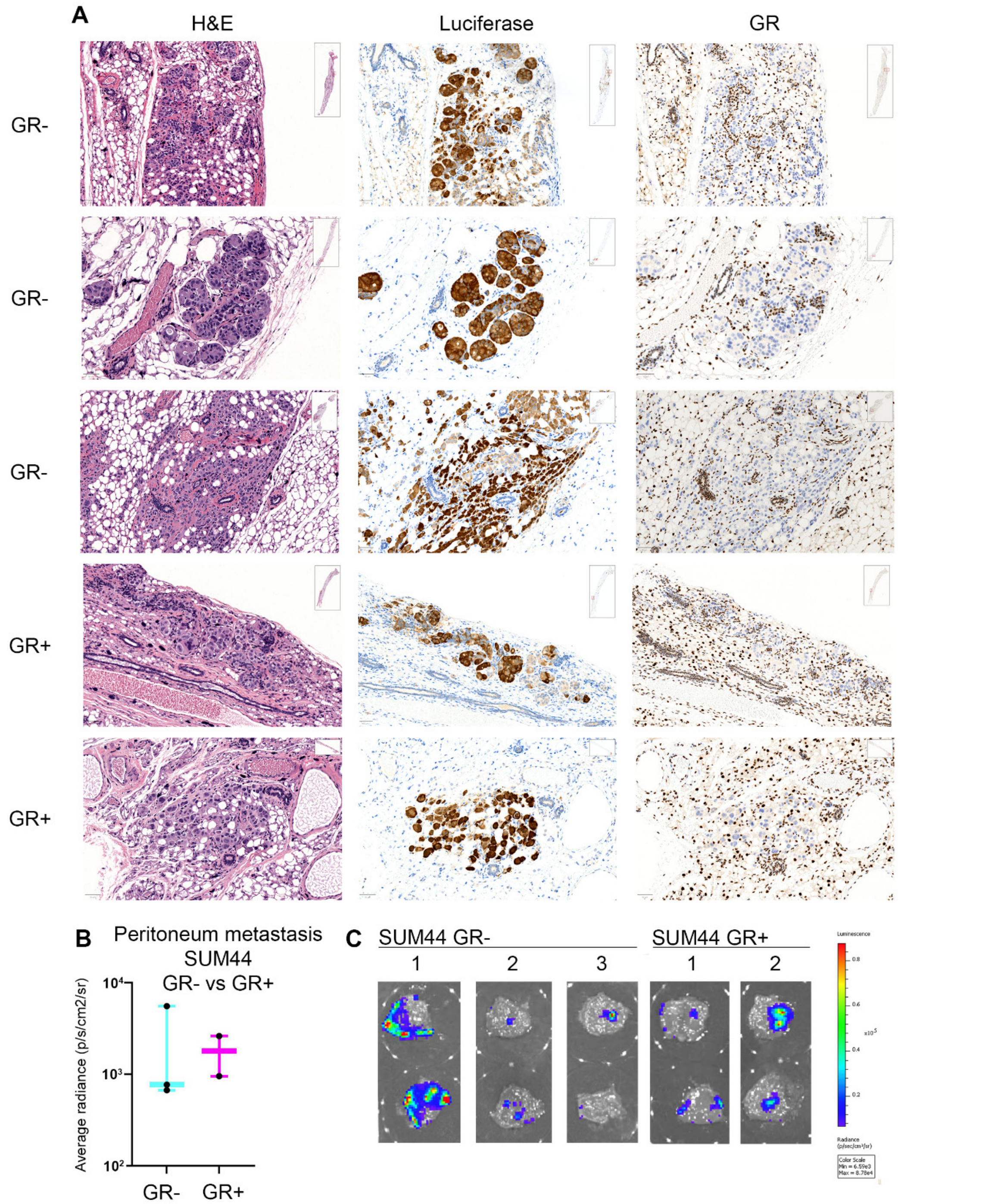

Figure S2. A) H&E, luciferase and GR IHC of GR- (n=3) and GR+ (n=2) representative primary tumors 113 days after intraductal injection SUM44 cells showing increased primary tumor size and distinct proliferative and invasive pattern of GR- tumors. B) Average radiance of peritoneum metastasis in SUM44 GR- and GR+ MIND models. C) Representative images of *ex* vivo bioluminescence of luciferase signal from peritoneum metastasis.
