# Supplementary figures and images for: Glucocorticoid Receptor Activation in Lobular Breast Cancer Is Associated with Reduced Cell Proliferation and Promotion of Metastases
Source: Cancers (Basel). 2023 Sep 22;15(19):4679. doi: 10.3390/cancers15194679 (PMC10571671; doi:10.3390/cancers15194679)

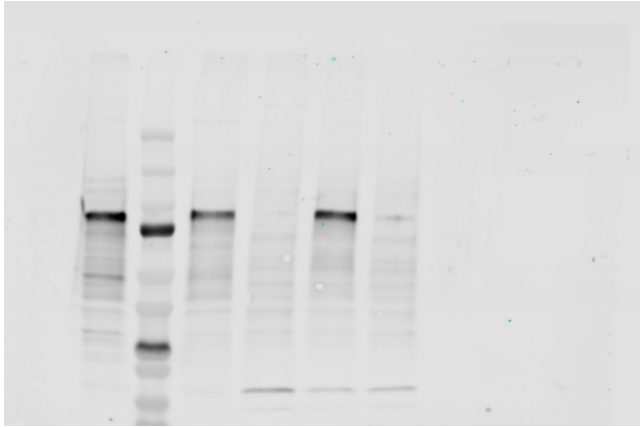

**Original image of Western blot**

Supplement: Supplementary file 1 [file cancers-15-04679-s001.zip › cancers-2557328-File S1.pdf]
